# CIN-RiskNet: a dynamic feature-enhanced TabTransformer with hybrid SMOTE-noise augmentation for contrast-induced nephropathy prediction

**DOI:** 10.3389/fcvm.2026.1909097

**Published:** 2026-08-10

**Authors:** Peng Zhang, Zehao Yang, Xue Zhang, Keyu Gong, Xiaogang Liu, Shicheng Yang, Zhiwei Zhang, Ximing Li, Runnan He

**Affiliations:** 1 Department of Cardiology, Chest Hospital, Tianjin University, Tianjin, China; 2 Tianjin Key Laboratory of Cardiovascular Emergency and Critical Care, Tianjin Municipal Science and Technology Bureau, Tianjin, China; 3 Medical School, Tianjin University, Tianjin, China; 4 Department of Cardiology, Central Hospital, Tianjin University, Tianjin, China; 5 Tianjin Key Laboratory of Ionic-Molecular Function of Cardiovascular Disease, Department of Cardiology, Tianjin Institute of Cardiology, the Second Hospital of Tianjin Medical University, Tianjin, China

**Keywords:** contrast-induced nephropathy, coronary heart disease, deep learning, percutaneous coronary intervention, self-attention, transformer

## Abstract

**Purpose:**

To propose a dynamic feature-enhanced TabTransformer framework that providing a more effective and accurate tool for predicting Contrast-Induced Nephropathy (CIN).

**Methods:**

This study proposes CIN-RiskNet, a dynamic feature-enhanced TabTransformer model integrated with a hybrid SMOTE-Noise augmentation strategy. The approach includes adaptive feature gating to suppress noise, synthetic minority oversampling to address class imbalance, and multi-head self-attention to capture complex feature interactions. The model was trained and evaluated under a leakage-free stratified five-fold cross-validation protocol, where SMOTE and Gaussian noise were applied only to the training split within each fold trained on a clinical dataset from Tianjin University Chest Hospital that including a total of 1,679 patients who underwent percutaneous coronary intervention for coronary heart disease.

**Results:**

Under leakage-free five-fold evaluation, CIN-RiskNet achieved strong performance with an accuracy of 95.40%, a recall of 95.40%, and an F1-score of 95.42%. It attained the highest F1-score and recall among all evaluated configurations. It outperformed not only traditional machine learning models including XGBoost, Random Forest, and Support Vector Machine, but also the Mehran risk score, a widely used clinical scoring system for CIN prediction. Ablation studies confirmed the contributions of each module, demonstrating improved robustness and generalization.

**Conclusion:**

The proposed model effectively addresses key challenges in CIN prediction, including class imbalance and feature noise, through an integrated deep learning framework. It shows promising potential as a decision-support tool, while external multicenter validation remains necessary before broad clinical deployment.

## 1 Introduction

Contrast-induced nephropathy (CIN) has emerged as a critical complication following invasive cardiovascular procedures, such as percutaneous coronary intervention (PCI), driven by the increasing global use of iodinated contrast media (CM) (1). CIN is defined as a significant increase in serum creatinine levels within 48 to 72 h after exposure to contrast media, representing a reversible yet dangerous form of acute renal failure (2). Studies have shown that patients who develop CIN after PCI have a significantly increased risk of adverse cardiovascular events, regardless of whether their renal function ultimately recovers. Especially among high-risk groups such as those with diabetes, the incidence rate of CIN can reach up to 50% (3). Currently, there is no effective treatment available, making early screening and prevention the only clinically effective strategy (4).

Risk prediction for CIN has long been a focal point of clinical research. Early methods for predicting CIN primarily relied on statistical models. For instance, Mehran identified eight independent risk factors using multiple regression and developed a CIN risk score (5). Simultaneously, with the continuous advancement of machine learning, methods such as random forests and ensemble models have been gradually introduced to enhance prediction accuracy (6–9). However, these methods are highly dependent on manual feature engineering, struggle to capture nonlinear relationships, and are highly sensitive to class imbalance issues.

Current research can effectively assist doctors in predicting the occurrence of CIN, but these methods still face two persistent challenges: Firstly, clinical datasets are filled with a large number of feature attributes and noise, requiring manual feature selection, which introduces a significant amount of subjective bias (10); Secondly, severe class imbalance leads to models favoring majority class predictions, thereby increasing the risk of missed diagnoses.

To address the issues, we propose CIN-RiskNet, a dynamic feature-enhanced TabTransformer framework. This model integrates the SMOTE-Noise enhancement strategy, which balances class distribution while simulating clinical measurement errors, and a dynamic feature gating mechanism that can adaptively suppress redundant information. The experimental results on the dataset from the Department of Cardiology at Tianjin University Chest Hospital demonstrate that CIN-RiskNet significantly outperforms traditional models in multiple evaluation metrics, including accuracy, precision, and recall, providing a more effective and accurate tool for predicting CIN.

## 2 Materials and methods

### 2.1 Clinical characterization of dataset

#### 2.1.1 Patients

To evaluate the performance of our proposed method, we utilized Tianjin University Chest Hospital Cardiology Department Dataset. This study consecutively enrolled patients who underwent PCI from 2023.04 to 2025.04. We conducted this study in accordance with the Declaration of Helsinki. Ethics approval was obtained from the Ethics Committee of Tianjin Chest Hospital (2021YS-031-01). All patients signed informed consent.

#### 2.1.2 Inclusion and exclusion criteria

Patients were eligible if they were 18 years of age or older, met the diagnostic criteria for coronary heart disease and subsequently undergo elective PCI. Exclusion criteria included: (1) acute renal failure or estimated Glomerular Filtration Rate (eGFR) < 30 mL/min/1.73 m^2^; (2) Severe heart failure [left ventricular ejection fraction (LVEF)] < 30% or B-type natriuretic peptide (BNP) > 5,000 ng/L) or cardiogenic shock; (3) Perform emergency PCI; (4) Have used contrast medium or are allergic to contrast medium within the past 7 days; (5) Primary kidney disease, long-term dialysis, or undergoing renal replacement therapy within 24 h after admission; (6) Severe liver and lung failure; (7) Abnormal coagulation function; (8) Infectious diseases; (9) Malignant tumor; (10) Death within 48 h of admission or hospital stay less than 48 h.

#### 2.1.3 Primary endpoint definition

The primary endpoint was occurrence of CIN, defined as a rise in serum creatinine (Scr) ≥ 44.2 μmol/L or ≥ 25% above baseline within 72 h after contrast medium administration (2). Baseline Scr was defined as the first serum creatinine value obtained within 24 h of hospital admission, before major surgery or specific nephrotoxic interventions, representing the initial renal function at admission. Postoperative Scr measurements were standardized across all included patients at 48 h and 72 h after the procedure. Sample collection and testing followed a uniform protocol. Cases with non-standard or missing key postoperative Scr time points were excluded to ensure temporal consistency and data reliability.

#### 2.1.4 Statistical analysis

Continuous variables were analyzed using Student's t-test and non-parametric tests, with outcomes presented as mean ± standard deviation (SD) or median and interquartile range, respectively. Categorical variables were assessed via Chi-square test and Fisher's exact test, and results were expressed as counts and percentages. All statistical analyses were performed using SPSS software (version 25.0; SPSS Inc., New York, USA).

#### 2.1.5 Dataset

**Figure 1:**
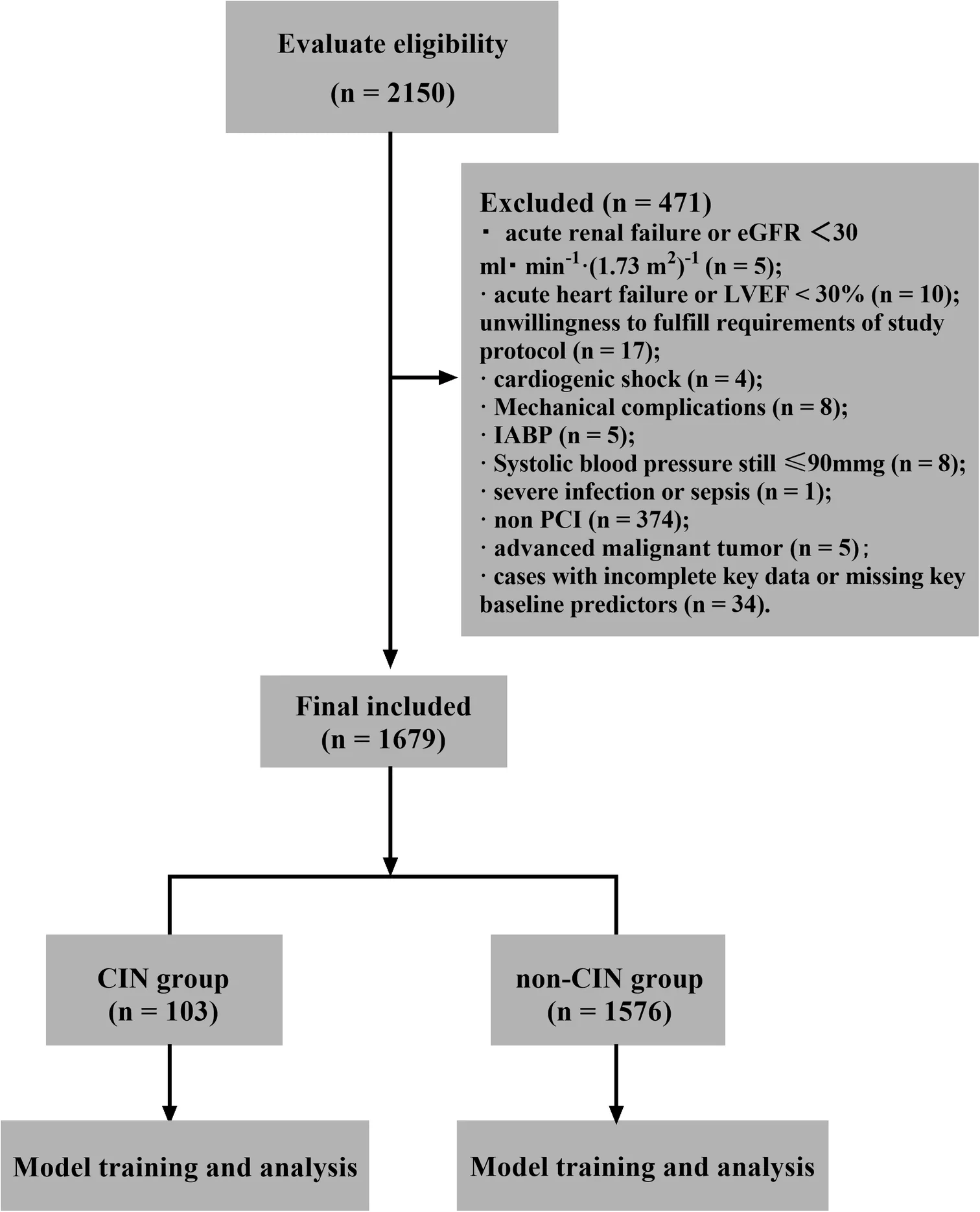
Flow diagram. CIN, contrast-induced nephropathy; eGFR, estimated glomerular filtration rate; IABP, intra-aortic balloon pump; LVEF, left ventricular ejection fraction; PCI, percutaneous coronary intervention.

Finally, a total of 1,679 patients who underwent PCI for coronary heart disease were finally included, among whom 103 were patients with CIN (Figure 1). The clinical baseline characteristics of the patients are presented in Table 1, a summary of its characteristics is provided in Table 2. We performed temporal feature governance and leakage prevention. To prevent outcome leakage, we predefined PCI/contrast administration as the prediction index time. Only variables available before or at this index time were eligible as model inputs. Post-procedural laboratory values, creatinine-change variables, and any endpoint-derived features were strictly excluded from model training, hyperparameter tuning, and SHAP interpretation. As shown in Table 3, only variables available before or at the index time were eligible as model inputs; hydration volume and all post-procedural creatinine-derived variables were excluded to prevent outcome leakage.

**Table 1:** Characteristics of the patients at baseline.

Variables	CIN group (*n* = 103)	non-CIN group (*n* = 1,576)	*P* value
Age (years)	67.7 ± 6.6	66.4 ± 7.3	0.189
Male (%)	69 (67.0)	867 (55.0)	0.021
BMI (kg/m^2^)	24.2 ± 3.3	24.7 ± 2.7	0.062
Mean LVEF	51.1 ± 7.2	53.3 ± 5.0	0.015
LVEF < 45% (%)	19 (18.6)	142 (9.0)	0.010
Hemoglobin (g/L)	122.6 ± 17.4	124.1 ± 19.3	0.489
*Medical history (%)*			
Prior PCI	16 (15.5)	205 (13.0)	0.397
Prior MI	13 (12.6)	161 (10.2)	0.285
Prior CHF	15 (14.6)	189 (12.0)	0.312
Hypertension	43 (41.7)	600 (38.1)	0.458
Hyperlipidaemia	49 (47.6)	683 (43.3)	0.369
Atrial fibrillation	5 (4.9)	32 (2.0)	0.064
Smoking	27 (26.2)	362 (23.0)	0.413
*Medications (%)*			
Aspirin	103 (100)	1,576 (100)	1
Clopidogrel	103 (100)	1,576 (100)	1
*β*-blockers	75 (72.8)	1,080 (68.5)	0.326
ACEI/ARB	63 (61.2)	1,066 (67.6)	0.189
Statins	98 (95.1)	1,538 (97.6)	0.157
Diuretics	28 (27.2)	378 (24.0)	0.402
Iopromide injection	103 (100)	1,576 (100)	1
*Surgical indication (%)*			
SAP	76 (73.8)	1,066 (67.6)	0.386
ACS	27 (26.2)	510 (32.4)	0.192
*Volume of contrast*			
<100 mL	15 (14.6)	477 (30.3)	0.001
100−199 mL	61 (59.2)	760 (48.2)	0.018
≥200mL	27 (26.2)	339 (21.5)	0.156
*Scr (μmol/L)*			
Baseline	76.2 ± 11.6	69.7 ± 13.4	0.001
Multivessel PCI (n ⩾2) (%)	43 (41.7)	550 (34.9)	0.047

ACS, acute coronary syndrome; ACEI, angiotensin-converting enzyme inhibitor; ARB, angiotensin receptor blocker; BMI, body mass index; CHF, congestive heart failure; LVEF, left ventricular ejection fraction; MI, myocardial infarction; PCI, percotaneous coronary intervention; SAP, stable angina pectoris, Scr, serum creatinine.

**Table 2:** Key variables of the Tianjin University Chest Hospital cardiology department dataset.

ID	Diabetes	ACEI	hs-CRP (mg/L)	Contrast dose (mL)	CIN
S01	0	1	0.45	150	0
S02	0	1	0.89	150	0
S03	1	1	0.24	50	1
S04	0	1	0.18	160	0
S05	1	1	0.33	220	0
S06	0	1	0.35	220	0

Diabetes (presence: 1 = yes, 0 = no), ACEI (angiotensin-converting enzyme inhibitor use: 1 = yes, 0 = no), hs-CRP (high-sensitivity C-reactive protein, mg/L), contrast dose (volume of iodinated contrast media administered, mL), and CIN (contrast-induced nephropathy diagnosis: 1 = positive, 0 = negative).

**Table 3:** Final candidate variables, acquisition timing, and leakage-control policy for CIN-riskNet.

Variable	Unit/coding	Acquisition timing
Sex	0 = female, 1 = male	Pre-PCI
Age	years	Pre-PCI
Height	cm	Pre-PCI
Weight	kg	Pre-PCI
BMI	kg/m^2^	Pre-PCI
Diabetes mellitus	0 = no, 1 = yes	Pre-PCI
Prior MI	0 = no, 1 = yes	Pre-PCI
LVEF	%	Pre-PCI
ACEI/ARB use	0 = no, 1 = yes	Pre-PCI
Diuretic use	0 = no, 1 = yes	Pre-PCI
CCB use	0 = no, 1 = yes	Pre-PCI
Compound Danshen dripping pills use	0 = no, 1 = yes	Pre-PCI
Triglycerides	mmol/L	Pre-PCI
Total cholesterol	mmol/L	Pre-PCI
HDL-C	mmol/L	Pre-PCI
LDL-C	mmol/L	Pre-PCI
β2-microglobulin	(as recorded)	Pre-PCI
ALT	U/L	Pre-PCI
hsCRP	mg/L	Pre-PCI
Contrast volume	mL	Intra-procedural
Preoperative Scr	μmol/L	Pre-PCI
Preoperative BUN	mmol/L	Pre-PCI
Preoperative eGFR	mL/min/1.73m^2^	Pre-PCI

ACEI, angiotensin-converting enzyme inhibitor; ARB, angiotensin receptor blocker; ALT, alanine aminotransferase; BMI, body mass index; BUN, blood urea nitrogen; CCB, calcium channel blocker; CHF, congestive heart failure; eGFR, estimated glomerular filtration rate; hsCRP, high-sensitivity C-reactive protein; LVEF, left ventricular ejection fraction; MI, myocardial infarction; PCI, percotaneous coronary intervention; Scr, serum creatinine.

### 2.2 Proposed method

**Figure 2:**
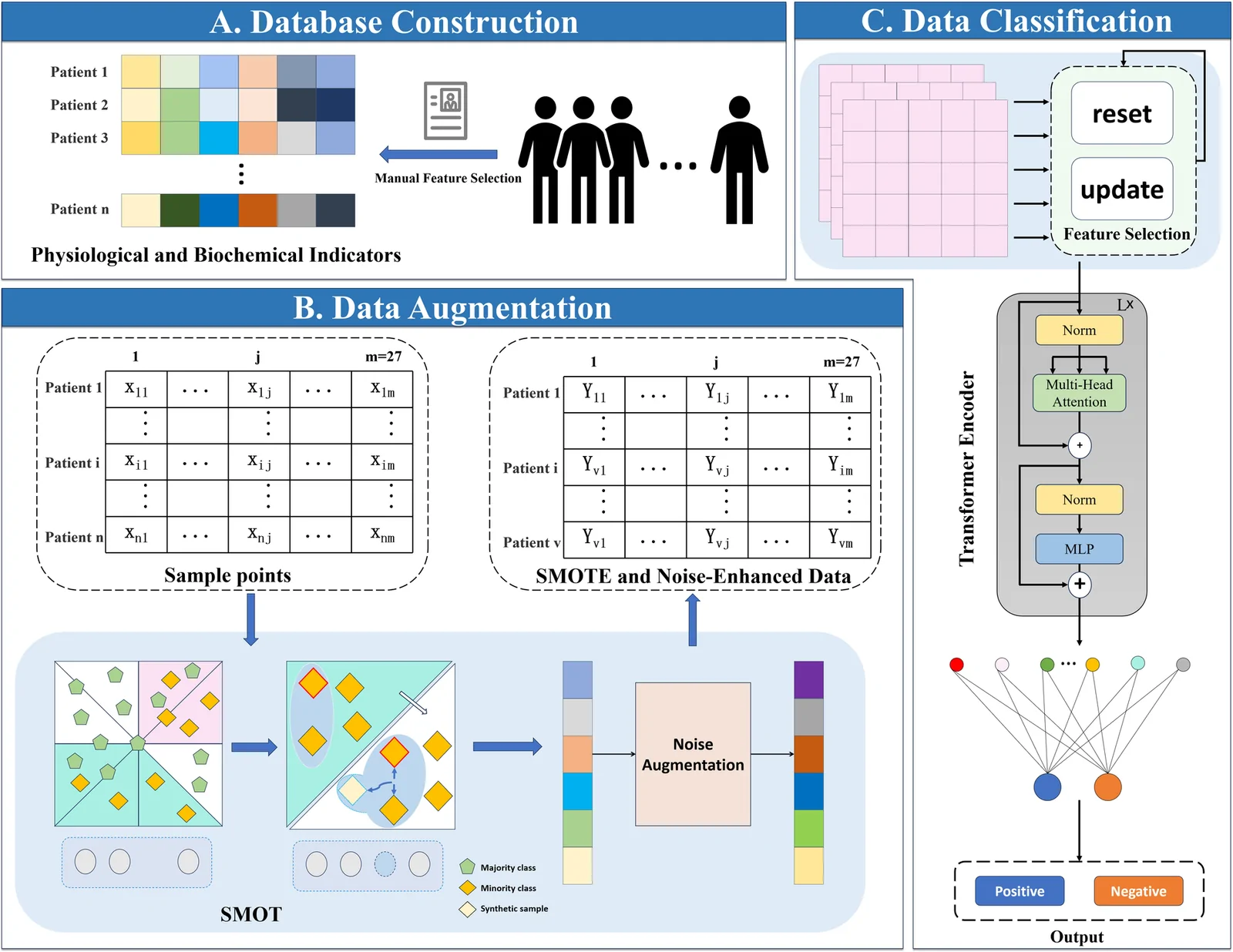
Overview of workflow. **(A)** Acquisition of multidimensional physiological and biochemical indicators from patient records. **(B)** Hybrid SMOTE-Noise data augmentation module: applying Synthetic Minority Oversampling Technique (SMOTE) to mitigate class imbalance and injecting calibrated noise to simulate clinical measurement variability, enhancing robustness. **(C)** Core predictive architecture: Gated feature selection dynamically filters informative features; a Transformer encoder with multi-head self-attention captures complex feature interactions; and a Multi-Layer Perceptron (MLP) head generates the final CIN risk prediction.

This study proposes the CIN-RiskNet designed for clinical tabular data. The core innovation lies in the integration of a dynamic feature gating mechanism with a TabTransformer encoder, enabling adaptive interaction modeling of multidimensional clinical features. This design addresses common challenges in medical data analysis, including noise sensitivity, class imbalance, and complex feature dependencies. The overall framework is illustrated in Figure 2.

Prior to feeding data into the model, we employed a SMOTE-Noise hybrid augmentation strategy to mitigate the effects of small sample size, high noise levels, and class imbalance inherent in clinical datasets. Specifically, SMOTE oversampling was first applied to the CIN-positive samples to generate synthetic instances and balance the positive/negative class ratio. To further prevent overfitting, Gaussian noise was randomly injected into both the synthetic and original samples.

The augmented dataset was then passed through the gating unit. Given that clinical data often contain a large number of low-informative or noisy features, traditional static feature selection approaches fail to adapt to patient-specific feature distributions. To address this, we adopted a gating mechanism for dynamic feature selection and representation learning. The gating unit assigns adaptive weights to input features, allowing the model to focus on clinically relevant variables while suppressing noise and redundancy.

**Figure 3:**
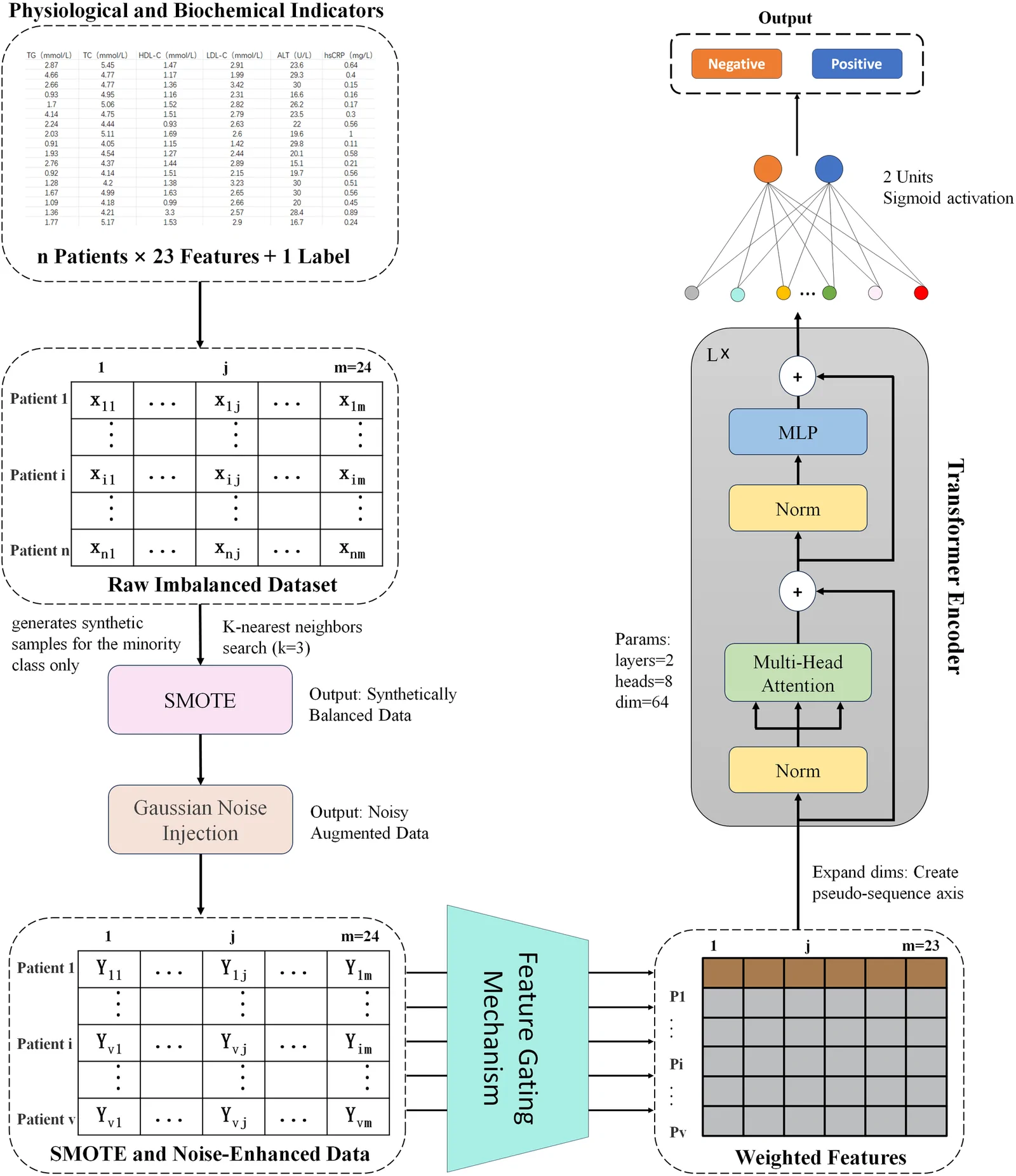
Architecture and workflow of the CIN-riskNet framework. Input layer: Tabular data of *n* patients structured as a *n* × 23 matrix of clinical measurements with corresponding binary labels. Processing pipeline: ① Hybrid Data Augmentation: SMOTE: Synthetic minority oversampling to balance class distribution. Gaussian Noise Injection: Simulates clinical measurement errors through feature-wise noise perturbation. ② Gated Feature Selection: Learns adaptive weights to suppress redundant/noisy features. ③ Transformer Encoder: Multi-head self-attention captures cross-feature interactions. Layer Normalization (Norm) stabilizes training. Multilayer Perceptron (MLP) refines representations. ④ Classification Head: Sigmoid-activated MLP outputting CIN risk probability. Output: Binary classification output: Negative (CIN risk <0.5)/Positive (CIN risk ≥0.5).

The output of the gating unit was subsequently fed into an embedding layer to obtain higher-dimensional representations, which were then passed into the TabTransformer encoder. Each of the 23 clinical variables was treated as an individual feature token. The input was represented as (batch,23,d), and self-attention was performed across feature tokens to model inter-feature dependencies. After processing through the encoder, the output was reduced in dimension to match the original feature space, followed by a multilayer perceptron (MLP)classifier that generated the final prediction. The whole architecture and workflow of the CIN-RiskNet framework is shown in Figure 3. The mathematical formulations of the proposed method are detailed in Equations 1–19.

### 2.3 SMOTE-noise hybrid augmentation strategy

Given the inherent class imbalance in medical datasets, resampling techniques can be employed to balance class distributions. One such method is undersampling, which creates a new dataset by removing samples from the majority class (11). Conversely, oversampling increases the number of minority class instances to approximate the size of the majority class, thereby generating a more balanced dataset. Since medical datasets typically suffer from both class imbalance and limited overall sample size, oversampling is generally more appropriate.

Synthetic Minority Oversampling Technique (SMOTE) (12) is a widely used and effective oversampling method that generates synthetic minority class samples by interpolating between existing instances and their nearest neighbors. Compared to undersampling, SMOTE often yields better model performance and is considered one of the most prevalent oversampling strategies across domains (13), including widespread use in medical data applications (14, 15).

**Figure 4:**
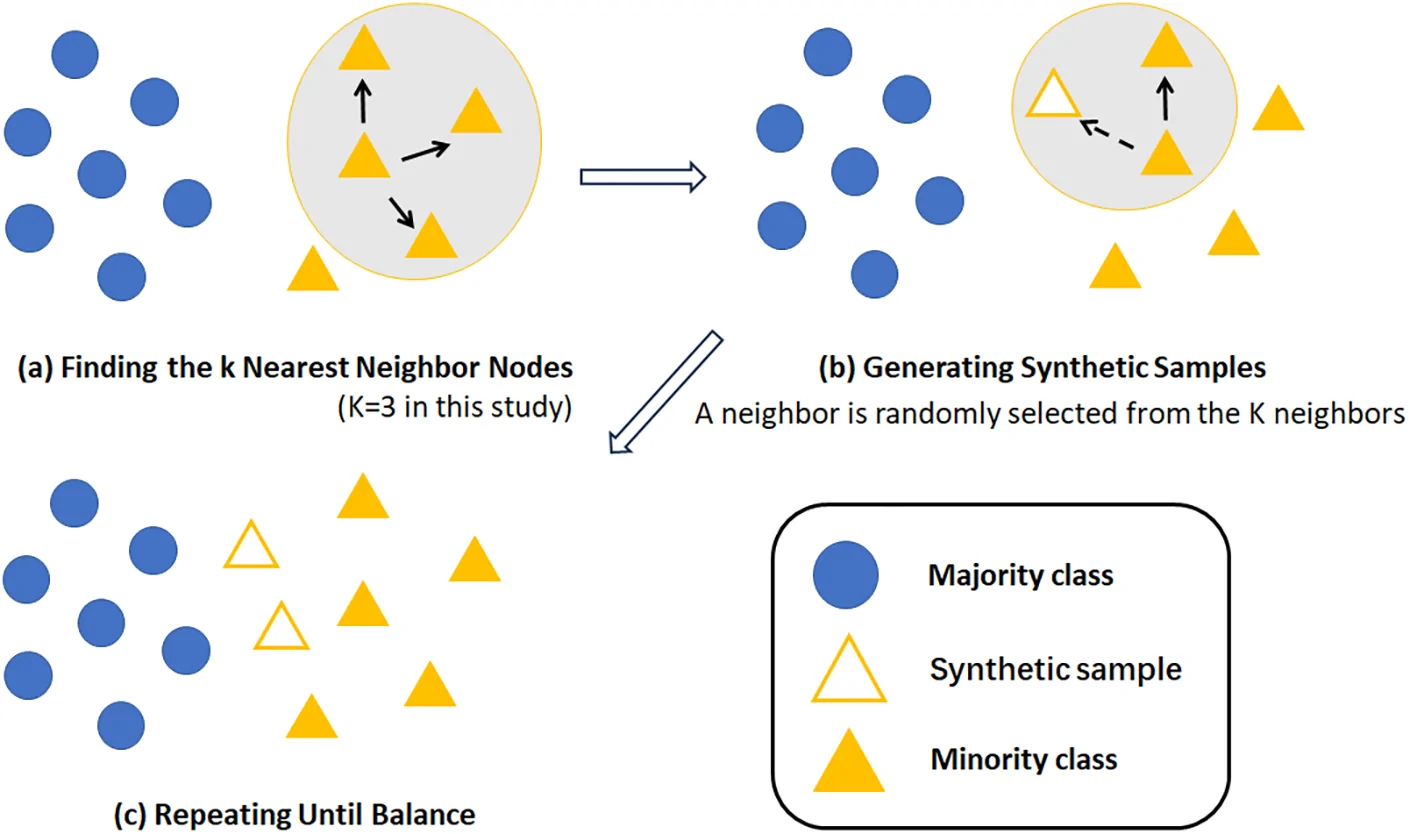
SMOTE algorithm flow diagram. **(a)** Neighbor Selection: For each CIN-positive patient xi, identify k = 3 nearest neighbors based on Euclidean distance. **(b)** Synthetic Generation: Randomly interpolate between xi and neighbor xj to create new samples xnew. **(c)** Output: Augmented training set with balanced class distribution.

In this study, SMOTE is applied to generate synthetic samples for the minority class—CIN-positive cases. The algorithm flow is illustrated in Figure 4. For each minority sample, SMOTE identifies its *K* nearest neighbors in the feature space, with *K* set to 3 in this study. Euclidean distance is used to measure the similarity between data points. After identifying the neighbors, SMOTE randomly selects one of them and computes the difference between the feature vectors of the minority sample and the selected neighbor. This difference is then multiplied by a random number between 0 and 1 and added to the feature vector of the minority sample, thereby generating a new synthetic sample. These synthetic samples lie along the line segments connecting the minority sample to its neighbors. This process is repeated until the desired level of class balance is achieved.

The number of synthetic samples generated via SMOTE is controlled by the imbalance ratio between the majority and minority classes. The oversampling ratio is defined as follows:

$$\begin{array}{c} N=\frac{\mathrm{Size}(x_{\mathrm{maj}})\text{-}\mathrm{Size}(x_{\min})}{\mathrm{Size}(x_{\min})}\;\times 100\text{\%} \end{array}$$ (1)

$$\begin{array}{c} \mathrm{Size}(x_{\mathrm{sy}n_{\min)}})=\mathrm{Size}(x_{\min})\;\times N \end{array}$$ (2)

where $Size(x_{maj})$ and $Size(x_{min})$ represent the sample sizes of the majority and minority classes, respectively. Based on the calculated oversampling ratio *N*, SMOTE determines the number of synthetic samples to generate $Size(x_{syn_{min}})$.

These samples are created by linear interpolation between a minority instance $X_{i}$ and one of its nearest neighbors $X_{j}$, as shown below:

$$\begin{array}{c} X_{\mathrm{syn}}=X_{i}+\lambda (X_{j}\text{-}X_{i}),\;\lambda ∼U(0,1) \end{array}$$ (3)

Here, $X_{i}$ is a minority-class sample, $X_{j}$ is one of its nearest neighbors, λ is a random interpolation coefficient drawn from a uniform distribution U(0,1), and $X_{syn}$ is the generated synthetic sample.

However, when the class imbalance is extreme, the large volume of synthetic samples may lead to high redundancy and overfitting. Additionally, such interpolated samples can be overly idealized and may not accurately reflect real-world variability. To address these limitations and simulate the measurement errors and data noise commonly observed in clinical scenarios, this study introduces a Gaussian noise-based hybrid augmentation strategy following SMOTE. The formula is as follows:

$$X^{\prime }=X+\epsilon \cdot N(0,I),\epsilon =0.01$$ (4)

Here, *X* denotes the original or synthetic feature vector, N(0,I) is multivariate Gaussian noise with zero mean and identity covariance matrix, and ϵ is a noise scaling factor that controls the degree of perturbation. The augmented data are subsequently fed into the feature gating unit for downstream processing.

### 2.4 Feature gating mechanism

The concept of gated units originated with the Long Short-Term Memory (LSTM) network (16), which uses a forget gate, an input gate, and an output gate to control information flow, allowing the network to selectively retain long-term information or discard irrelevant data, thereby addressing the vanishing and exploding gradient problems of traditional recurrent neural network. Building on LSTM, Chung et al. introduced the Gated Recurrent Unit (GRU) (17) in 2014, which simplifies the gating structure by merging LSTM's three gates into two (reset gate and update gate) and eliminating the cell state in favor of relying solely on the hidden state for information propagation. Gating mechanisms have been widely applied in domains such as speech recognition (18) and image processing (19), and have also been reported in medical applications (20). It serves as a dynamic feature selection technique, designed to apply learnable weight matrices to adaptively emphasize informative features and suppress noisy or redundant ones. Clinical tabular data typically contain numerous low-informative features and significant noise. Traditional static feature selection approaches, which rely on manual screening or fixed weighting schemes, often fail to adapt to the heterogeneous feature distributions across different patients. To address this issue, we incorporate a dynamic feature gating module based on a trainable gating weight matrix. After the original input undergoes SMOTE-Noise augmentation, the enhanced data are passed into the gating unit, computed as follows:

$$\begin{array}{c} G=\sigma (W_{g}X+b_{g}) \end{array}$$ (5)

$$\begin{array}{c} X^{\prime }=X⊙G \end{array}$$ (6)

Here, σ denotes the sigmoid activation function， *X* represents the input feature vector, $W_{g}\in R^{d\times d}$ and $b_{g}\in R^{d}$ are trainable parameters, and ⊙ indicates element-wise multiplication. This mechanism generates a feature importance score for each input dimension via linear transformation followed by a non-linear gating operation. The resulting weighted feature representation $X^{\prime }$ is obtained by element-wise multiplication of the original input with the learned gate vector. All gating parameters are optimized through backpropagation, enabling the model to learn a task-specific feature selection strategy in an end-to-end manner.

Through this mechanism, the model automatically enhances features that are critical for CIN prediction while suppressing irrelevant or noisy dimensions. The dimensionality of the output from the gating unit remains the same as the input and is subsequently fed into the embedding layer.

In this study, each sample in the Tianjin University Chest Hospital Cardiology Department Dataset contains 23 clinical features. The input batch size is set to 32, and after processing through a DataLoader, each input batch is represented as a tensor of shape $X_{in}\in R^{32\times 23}$.The embedding layer transforms this input from a 23-dimensional space to a 64-dimensional space, using the following operation:

$$\begin{array}{c} X_{\mathrm{embed}}=X_{\mathrm{gate}}\cdot W_{e}+b_{e} \end{array}$$ (7)

Here $X_{embed}\in R^{32\times 64}$. $W_{e}$ and $b_{e}$ are the trainable weight matrix and bias vector, respectively. The embedded feature representation is then forwarded to the TabTransformer encoder for further modeling.

### 2.5 Training and testing strategy

The Transformer network, initially proposed by Vaswani et al. for machine translation, introduced the groundbreaking self-attention mechanism, which dynamically calculates the pairwise dependencies between elements in a sequence, granting the model strong global contextual awareness. While the Transformer has achieved state-of-the-art results in fields such as image recognition and natural language processing, it has shown limitations in handling tabular data when compared to conventional machine learning models (21, 22). TabTransformer addresses these limitations through targeted modifications for structured tabular data. Unlike the standard Transformer, TabTransformer introduces the following key adaptations: (1) Removal of positional encoding: Tabular features are typically unordered and do not follow a natural sequential structure. (2) Feature-wise embedding: Each column (feature) is individually mapped to a dense vector to capture semantic relationships between features. (3) Context-aware attention: Self-attention models nonlinear inter-feature dependencies, replacing the need for manually designed feature crosses.

In this study, we adopt only the encoder module of the TabTransformer for the CIN classification task, as it effectively captures global inter-feature interactions without requiring a decoder. The encoder consists of multi-head self-attention (MSA) and a feed-forward network (FFN). MSA splits the input feature space into multiple subspaces (attention heads), with each head learning a distinct interaction pattern. Self-attention operates by computing similarity scores between query (Q) and key (K) pairs and applying these weights to the value (V) representations. The computation is defined as:

$$\begin{array}{c} \mathrm{MultiHead}(Q,K,V)=\mathrm{Concat}(\mathrm{hea}d_{1},\;\mathrm{ldots},\mathrm{hea}d_{h})W^{O} \end{array}$$ (8)

$$\begin{array}{c} \mathrm{hea}d_{i}=\mathrm{Softmax}\left(\frac{{\mathrm{QW}}_{i}^{Q}{{(\mathrm{KW}}_{i}^{K})}^{T}}{\sqrt{d_{k}}}\right){\mathrm{VW}}_{i}^{V} \end{array}$$ (9)

Here, Q,K,V are projections of the input, and $W_{i}^{Q},W_{i}^{K},W_{i}^{V}\in R^{n\times d}$ are the learned projection matrices for the i-th attention head. $W^{O}\in R^{hd_{v}\;\times d}$ projects the concatenated output back to the desired dimension. The number of heads h is set to 8, and the feature dimension d=64 with a scaling factor $\sqrt{d_{k}}$ to stabilize gradients.

Although tabular data lack a natural sequence structure, TabTransformer requires a sequence dimension in its input. In the revised TabTransformer design, each of the 23 clinical variables is treated as an individual feature token rather than collapsing all variables into a single token, and then reshaping the input tensor as $X_{\mathrm{unsqueezed}}\in R^{32\times 23\times 64}$.

The encoder is composed of L=2 identical layers, each containing an MSA sub-layer and an FFN sub-layer. Every sub-layer is followed by residual connections and layer normalization, as defined by:

$$\begin{array}{c} S_{i}^{{}^{\prime }}=\mathrm{MSA}(S_{i\text{-}1})+S_{i\text{-}1} \end{array}$$ (10)

$$\begin{array}{c} S_{i}^{{}^{\prime }}=\mathrm{LayerNorm}(S_{i}^{{}^{\prime }}) \end{array}$$ (11)

$$\begin{array}{c} S_{i}=\mathrm{FFN}(S_{i}^{{}^{\prime }})+S_{i}^{{}^{\prime }} \end{array}$$ (12)

$$\begin{array}{c} S_{i}=\mathrm{LayerNorm}(S_{i}) \end{array}$$ (13)

Each FFN module comprises two linear layers with a ReLU activation between them. The hidden dimension is expanded to four times the original size and then projected back to the original dimension:

$$\begin{array}{c} \mathrm{FFN}(X)=W_{2}\cdot \mathrm{ReLU}(W_{1}\cdot X+b_{1})+b_{2} \end{array}$$ (14)

Where $W_{1}\in R^{64\times 256},\;W_{2}\in R^{256\times 64}$.

The output of the final encoder layer is flattened (removing the dummy sequence dimension) and passed into a single-layer fully connected classification head, transforming the 64-dimensional embedding into a 2-dimensional class probability vector:

$$\begin{array}{c} \hat{y}=\mathrm{Softmax}(X_{\mathrm{squeezed}}\cdot W_{c}+b_{c}) \end{array}$$ (15)

Where $\hat{y}\in R^{32\times 2}$, and $X_{\mathrm{squeezed}}\in R^{32\times 64}$. The unsqueeze and squeeze operations are purely for tensor reshaping, and do not alter the content of the data—only its shape, to match the model's interface requirements.

### 2.6 Model training and evaluation protocol

This study employs five-fold cross-validation, where the entire patient dataset is randomly divided into five mutually exclusive subsets. In each iteration, one subset is selected as an independent test set, while the remaining four are combined to form the training set. Importantly, in each fold, SMOTE and Gaussian noise were applied strictly to the training subset only, while validation and test subsets were kept untouched to prevent synthetic-sample leakage. This process is repeated five times to ensure that every data instance is used for testing. In each experiment, the training and validation sets are strictly non-overlapping, ensuring that the validation results reflect the model's generalization performance on unseen data. Furthermore, validation based on five distinct data splits mitigates potential biases arising from chance-based data partitioning, thereby enhancing the reliability of experimental conclusions. Within the training set, 20% of the samples are further separated as a validation set, and the remaining 80% are used for model training to prevent information leakage.

The model is trained for 75 epochs with a batch size of 32, using the Adam optimizer (initial learning rate of 0.001 and weight decay of 1 × 10⁻⁵), minimizing the cross-entropy loss. Our model is implemented in Python 3.9 and Torch 2.5, and is configured to run on a GeForce RTX 2080 Ti GPU.

### 2.7 Comparison with clinical scoring

Mehran score is a widely used clinical risk stratification tool for CIN prediction. However, several variables required by the original Mehran score (e.g., hypotension, intra-aortic balloon pump use, congestive heart failure) were not routinely documented in our structured electronic health records, a common limitation in retrospective studies. We therefore constructed a modified Mehran-like comparator using available variables (age, diabetes, contrast volume, baseline Scr/eGFR) to provide a pragmatic local benchmark.

We emphasize that this comparison is intended solely as a supplementary clinical reference, not as a primary evaluation metric. Our principal comparative assessment relies on multiple machine learning baselines (LOG, RF, XGBoost, SVM, MLP) trained under identical data partitions and preprocessing pipelines (Table 4), where CIN-RiskNet demonstrated consistent superiority. The modified Mehran comparator is included to contextualize model performance within our institutional data environment, acknowledging that structured datasets often lack variables required by established risk scores.

**Table 4:** Comparison of the performance of the models on the test set.

Model	Accuracy	Precision	Recall	F1-score
LOG	0.7167	0.1337	0.6417	0.2213
RF	0.9621	1.0000	0.3992	0.5706
XGBoost	0.9588	0.9156	0.3992	0.5560
SVM	0.9483	0.7889	0.2533	0.3835
MLP	0.9443	0.5667	0.1275	0.2082
Our method	0.9540	0.9545	0.9540	0.9542

LOG, logistic regression; RF, random forest; SVM, support vector machine; MLP, multilayer perceptron. Metrics range: [0,1] (higher values indicate better performance).

## 3 Results

To evaluate the performance of the proposed method, we conducted a quantitative analysis using commonly used evaluation metrics:

- **Accuracy** reflects the overall proportion of correctly predicted samples:
  $$\begin{array}{c} \mathrm{Accuracy}=\frac{\mathrm{TP}+\mathrm{TN}}{\mathrm{TP}+\mathrm{TN}+\mathrm{FP}+\mathrm{FN}}\; \end{array}$$ (16)

**Precision** measures the proportion of correctly predicted CIN-positive cases among all predicted positives. High precision ensures reliable identification of high-risk patients, reducing unnecessary interventions:

$$\begin{array}{c} \mathrm{Precision}=\frac{\mathrm{TP}}{\mathrm{TP}+\mathrm{FP}} \end{array}$$ (17)

- **Recall** quantifies the proportion of actual CIN-positive cases correctly identified. High recall aligns with the medical ethical principle of “prioritizing reduced missed diagnoses,” especially critical for high-risk complications like CIN:
  $$\begin{array}{c} \mathrm{Recall}=\frac{\mathrm{TP}}{\mathrm{TP}+\mathrm{FN}} \end{array}$$ (18)

**F1-score** balances precision and recall via their harmonic mean. For imbalanced datasets, F1 provides a more robust evaluation than individual metrics:

$$\begin{array}{c} F1=\frac{2\;\mathrm{timesPrecision}\;\mathrm{timesRecall}}{\mathrm{Precision}+\mathrm{Recall}} \end{array}$$ (19)

Where: TP (True Positive) refers to patients who are actually CIN-positive and are correctly predicted as positive by the model. TN (True Negative) refers to patients who are actually CIN-negative and are correctly predicted as negative. FP (False Positive) refers to patients who are actually CIN-negative but are incorrectly predicted as positive by the model. FN (False Negative) refers to patients who are actually CIN-positive but are missed (predicted as negative) by the model.

We compared the proposed model against mainstream classifiers, including logistic regression (LOG) (23), random forest (RF) (24), support vector machine (SVM) (25), XGBoost (26) and MLP.

**Figure 5:**
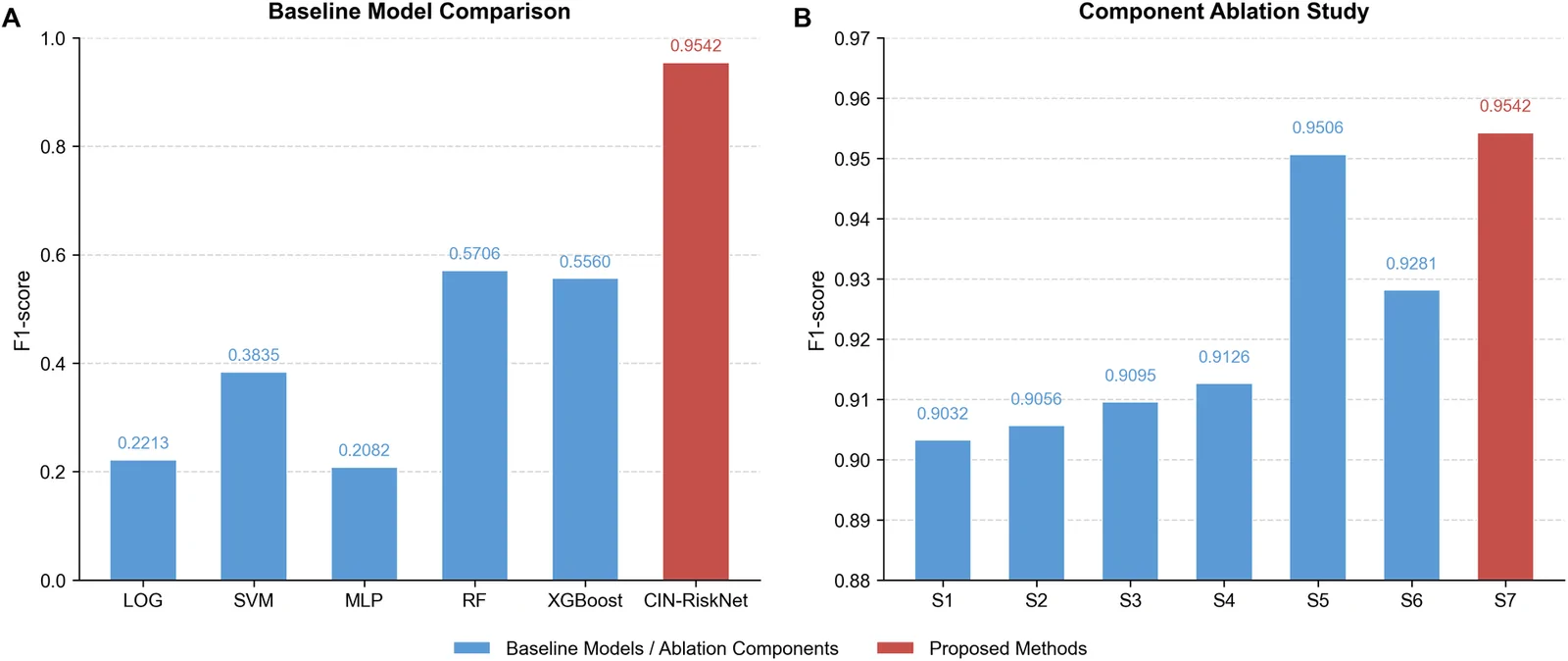
Performance analysis of CIN-riskNet for contrast-induced nephropathy prediction. **(a)** Baseline model comparison: Performance of CIN-RiskNet versus clinical prediction models. **(b)** Component ablation study: Contribution of each innovation to the final performance. Data source: Tianjin University Chest Hospital Cardiology Department | Metric: F1-score.

To ensure fairness in model comparisons, all methods adopted identical training/testing splits, uniform data preprocessing pipelines, and 5-fold cross-validation. Comparative performance across four key metrics (accuracy, precision, recall, and F1-score) is detailed in Table 4 and visually highlighted in Figure 5(a).

Among all compared models, CIN-RiskNet achieved the highest recall (0.9540) and F1-score (0.9542), substantially outperforming traditional machine learning baselines in detecting minority-class samples (Table 4). Although RF and XGBoost attained marginally higher accuracy (0.9621 and 0.9588) and exhibited strong precision (1.0000 and 0.9156), their recall was severely compromised (0.3992 for both), indicating that these ensemble methods would miss over 60% of true CIN cases—a clinically unacceptable trade-off in screening contexts where false negatives carry severe consequences. By contrast, CIN-RiskNet maintained high precision (0.9545) without sacrificing sensitivity, reflecting robust adaptive feature interaction modeling rather than reliance on manually engineered feature interactions. SVM exhibited extreme precision–recall imbalance (0.7889 and 0.2533), yielding a low F1-score (0.3835) that confirms its unsuitability for imbalanced clinical tasks prioritizing reduced missed diagnoses. LOG and MLP delivered poor overall performance (F1-scores of 0.2213 and 0.2082), constrained by linear separability assumptions or insufficient and unstable capacity to model global feature dependencies. Collectively, these findings validate the superior sensitivity and robust classification capability of CIN-RiskNet across the entire dataset.

The proposed model's F1-score of 0.9542 highlights its exceptional balance between precision and recall—critical for clinical applications requiring simultaneous minimization of false positives (avoiding unnecessary interventions) and false negatives (preventing missed diagnoses). Further validating the effectiveness of the dynamic gating mechanism and SMOTE-Noise hybrid strategy in addressing class imbalance and noise sensitivity. These results solidify the model's potential as a robust tool for high-risk CIN prediction.

In summary, the proposed model achieved state-of-the-art performance, surpassing traditional machine learning approaches across all key metrics—accuracy, precision, recall, and F1-score—with its efficacy further demonstrated by stable training convergence and low classification error rates. These performance estimates should be interpreted in the context of internal validation within a single-center retrospective cohort. Although leakage control was strictly enforced, transportability across different institutions, assay platforms, and case-mix distributions remains to be verified.

It can be seen that the prediction ability of the model is indeed strong, while we cannot ignore the preconditions of imbalanced data samples. This is an unavoidable limitation of this study, but the observed gains from imbalance sample distribution should be interpreted in the clinical context of CIN prevalence. In real-world PCI cohorts, CIN events are relatively infrequent compared with non-CIN cases, resulting in an inherently imbalanced outcome distribution. Therefore, improvements from SMOTE-based rebalancing are expected and clinically meaningful, particularly for reducing missed high-risk cases while maintaining overall discrimination and calibration under a leakage-free evaluation protocol.

**Figure 6:**
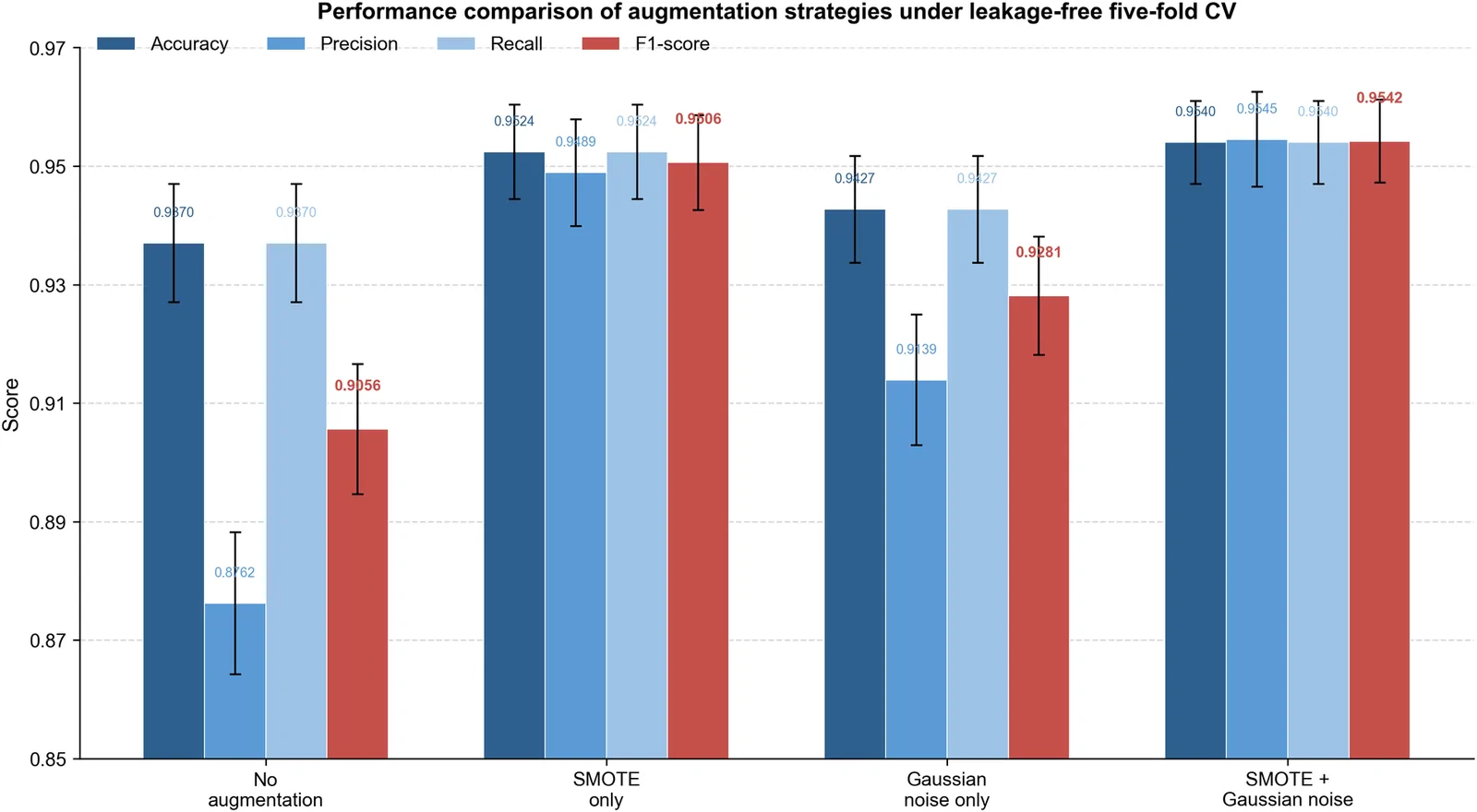
Performance comparison of augmentation strategies under leakage-free five-fold CV. Bar chart of classification metrics (accuracy, precision, recall, and F1-score) for four augmentation settings under identical model architecture and training configuration: no augmentation, SMOTE only, Gaussian noise only, and SMOTE + Gaussian noise. Error bars indicate fold-wise variability. Compared with the non-augmentation baseline, all augmentation strategies improved performance, with hybrid SMOTE-Noise settings showing consistently higher scores across all reported metrics.

Under the five-fold cross-validation setting, we compared three augmentation strategies using identical architecture and training parameters: (1) no augmentation, (2) SMOTE only, (3) Gaussian noise only and (4) SMOTE combined with Gaussian noise. The baseline model without augmentation achieved an accuracy of 0.9370 and an F1 score of 0.9056. Applying SMOTE alone substantially improved the model's performance, increasing the accuracy to 0.9524 and the F1 score to 0.9506. Notably, the combination of SMOTE and Gaussian noise yielded the highest predictive performance, reaching an accuracy of 0.9540 and an F1 score of 0.9542. Under leakage-free five-fold cross-validation, SMOTE combined with Gaussian noise achieved the highest F1-score (0.9542) and recall (0.9540) among all augmentation configurations. SMOTE alone produced a slightly lower F1-score (0.9506), confirming that the hybrid strategy optimally balances precision and recall for CIN prediction. These results establish SMOTE combined with Gaussian noise as the optimal augmentation strategy, achieving superior performance across all key metrics. The comparative performance of the four augmentation settings is illustrated in Figure 6.

Importantly, these improvements were obtained under strict leakage-free stratified cross-validation, where augmentation was applied only to the training subset in each fold.

To provide a clinically interpretable benchmark, we compared CIN-RiskNet with a modified Mehran comparator under the same stratified five-fold test splits. CIN-RiskNet achieved a markedly higher AUROC (0.7594 vs. 0.5094). The near-random discrimination of the modified Mehran comparator reflects the substantial missingness of key clinical variables in our structured dataset—notably hypotension, intra-aortic balloon pump use, and congestive heart failure—rather than intrinsic inadequacy of the original Mehran score. This underscores a practical challenge in retrospective studies: structured electronic health records often lack variables required by established risk scores, limiting the direct applicability of traditional scoring systems.

Although the Brier score of CIN-RiskNet was modestly higher (0.1178 vs. 0.0639), this is expected given the trade-off between strong discrimination and marginal probability calibration: a comparator with near-random discrimination tends to predict close to the baseline event rate for most patients, yielding a deceptively low Brier score that reflects clinical non-informativeness rather than true calibration superiority. Importantly, CIN-RiskNet's superiority is independently confirmed by its consistent outperformance of all machine learning baselines (Table 4), which were evaluated under identical data and validation conditions without reliance on clinical scoring variables. Collectively, these findings support the incremental predictive utility of CIN-RiskNet for clinical risk stratification, particularly in settings where traditional risk scores cannot be fully reconstructed from available data.

To further validate the performance and feasibility of individual modules, we conducted ablation studies by systematically removing or replacing components of the proposed method. Additionally, we integrated multiple alternative modules with the baseline TabTransformer for comparative analysis. The comprehensive results of these ablation and module substitution experiments are documented in Table 5 and visually presented in Figure 5(b).

**Table 5:** Ablation study.

Model	Precision	Recall	F1-score
S1-Transformer	0.8731	0.9355	0.9032
S2-5-fold CV	0.8762	0.9370	0.9056
S3-Gating mechanism	0.8805	0.9405	0.9095
S4-CLS pooling	0.8845	0.9425	0.9126
S5-SMOTE	0.9489	0.9524	0.9506
S6-Noise (without SMOTE)	0.9139	0.9427	0.9281
Our method	0.9545	0.9540	0.9542

As shown in Table 5, when the original Transformer was directly applied to medical tabular data with a single random split (S1), it achieved an accuracy of 0.9355, a precision of 0.8731, and an F1-score of 0.9032. Its lack of optimization for feature independence and the single-split evaluation made it sensitive to data distribution shifts, particularly in the minority class. Through 5-fold cross-validation (S2), the model's accuracy improved to 0.9370 (+0.15%), while the F1-score increased from 0.9032 to 0.9056, demonstrating effective mitigation of data distribution bias and more stable performance estimation.

The dynamic gating mechanism (S3) further elevated the F1-score from 0.9056 to 0.9095 (+0.39%), with precision rising from 0.8762 to 0.8805. This confirms that the gating mechanism is a core contributor to model robustness, enhancing the model's ability to selectively emphasize clinically relevant features. Replacing mean pooling with a CLS token (S4) yielded marginal gains (F1-score 0.9095 to 0.9126), suggesting that the dynamic gating mechanism already captures sufficient global context.

Notably, integrating SMOTE (S5) produced the most substantial improvement: precision surged from 0.8845 to 0.9489 (+6.44%), and the F1-score improved from 0.9126 to 0.9506 (+4.16%). This indicates that class imbalance was the primary bottleneck in CIN prediction, and synthetic minority oversampling effectively compensated for the scarcity of positive cases. In contrast, noise augmentation alone (S6) improved precision to 0.9139 and F1 to 0.9281, but its effect was markedly weaker than SMOTE, suggesting that insufficient minority-class samples, rather than simple overfitting, was the dominant issue.

Combining SMOTE with Gaussian noise augmentation (S7) yielded consistent performance improvements across all key metrics. Accuracy increased from 0.9524 to 0.9540, precision from 0.9489 to 0.9545, recall from 0.9524 to 0.9540, and the corrected F1-score from 0.9506 to 0.9542. These results demonstrate that the hybrid SMOTE-Noise strategy achieves superior precision-recall balance compared to SMOTE alone, with the Gaussian noise component enhancing robustness to clinical measurement variability without compromising discriminative power. The complementary mechanisms, SMOTE addressing class imbalance through synthetic minority expansion and Gaussian noise simulating real-world measurement errors, jointly optimize model performance for CIN screening applications. Consequently, the configuration leveraging cross-validation, dynamic gating, and SMOTE combined with Gaussian noise emerges as the optimal framework for CIN prediction.

**Figure 7:**
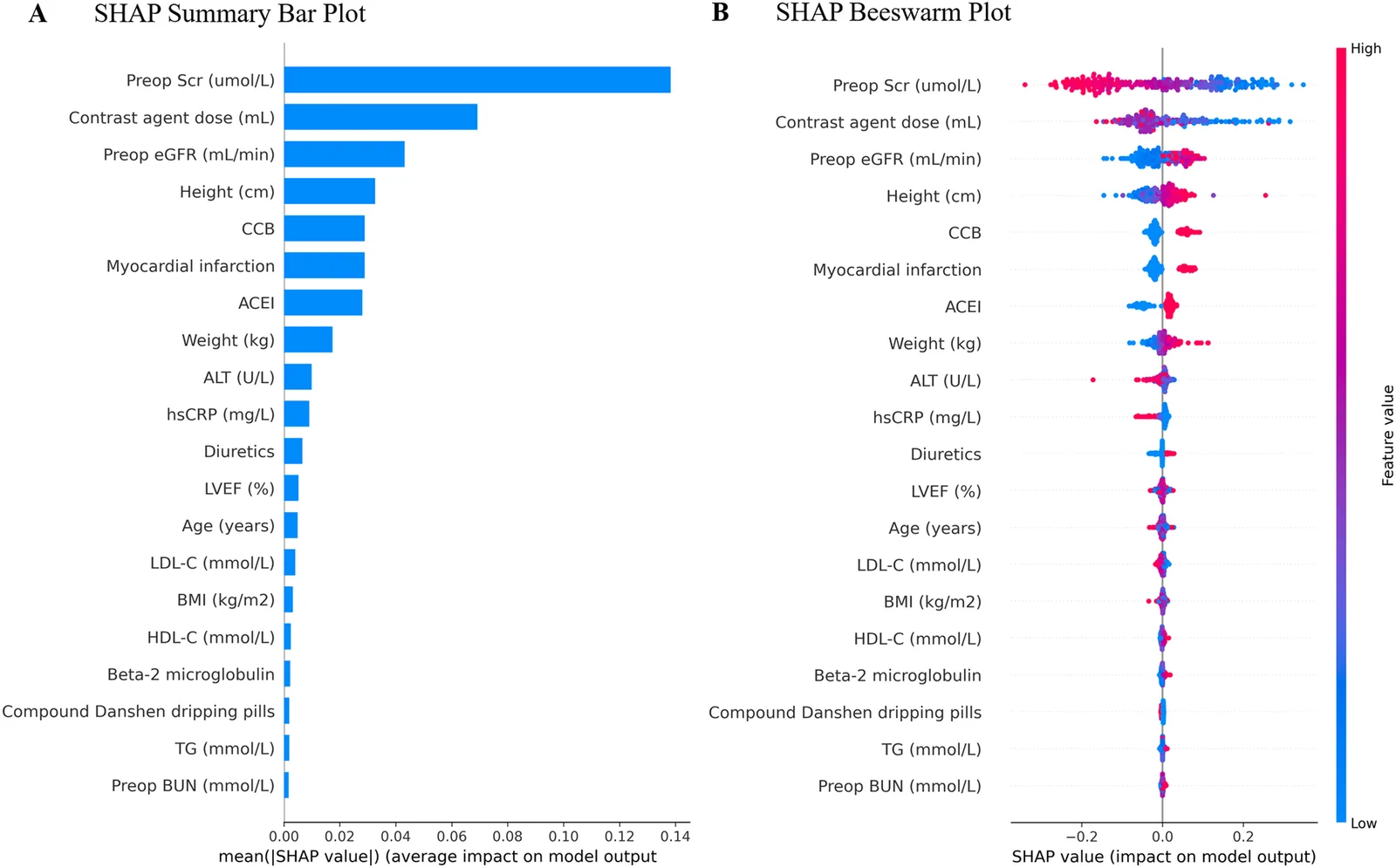
SHAP-based interpretability analysis of CIN-riskNet. **(A)** SHAP summary bar plot ranking global feature importance by mean absolute SHAP value. Features with larger values contribute more strongly to model predictions at the population level. **(B)** SHAP beeswarm plot showing the distribution and direction of feature effects across individual patients. Each dot represents one sample; the *x*-axis indicates SHAP value (negative to positive impact on model output), and color encodes feature magnitude (blue = low, red = high). Across both panels, renal-function-related variables and clinically relevant factors are among the most influential predictors, supporting clinical plausibility of the model's decision process.

To improve interpretability, we conducted SHAP analysis on the revised CIN-RiskNet and reported both a SHAP summary bar plot and a SHAP beeswarm plot. As shown in Figure 7A (bar plot) and Figure 7B (beeswarm plot), the model's predictions were predominantly driven by renal-function-related variables and clinically relevant treatment and risk factors. All SHAP analyses were performed using the final leakage-controlled feature set containing only pre-procedural/intra-procedural variables.

Specifically, the top features demonstrated clear physiological relevance. Elevated preoperative serum creatinine and reduced eGFR strongly increased the predicted risk, as they directly reflect diminished baseline glomerular filtration reserve—the most established predisposing factor for CIN. Larger contrast agent doses also markedly amplified risk, consistent with the dose-dependent tubular toxicity and medullary hypoxia induced by iodinated contrast media; higher volumes prolong renal exposure and overwhelm the kidneys’ compensatory mechanisms. Height emerged as a notable predictor, likely serving as a proxy for body surface area and thereby modulating the effective contrast concentration relative to renal cortical mass.

Among the remaining influential variables, a history of myocardial infarction and the use of CCBs and ACEIs showed appreciable positive contributions to predicted risk. These associations likely reflect underlying hemodynamic instability and compromised renal microcirculation in patients with advanced cardiovascular disease, rather than direct nephrotoxicity of the medications themselves. Notably, diuretic use, reduced LVEF, and advanced age exhibited relatively modest SHAP values, suggesting limited independent predictive leverage beyond renal function and contrast exposure within this strictly preoperative prediction framework. Conversely, metabolic indicators such as lipid profiles, BMI, and preoperative BUN demonstrated minimal influence on model output.

These findings indicate that the model focuses on biologically plausible predictors and is consistent with known CIN risk mechanisms. The most influential predictors were mainly renal-function-related and clinically meaningful variables, suggesting that the model’s decision process is aligned with known CIN risk mechanisms.

## 4 Discussion

As a major contributor to hospital-acquired acute kidney injury, CIN poses a serious threat to patient safety, carrying substantial risks of complications and mortality for those already at high risk (27). The underlying mechanisms involve direct tubular toxicity and medullary ischemia induced by iodinated contrast media, particularly when renal functional reserve is already compromised (28, 29). Although the Mehran score provides a contemporary risk stratification tool, external validation studies report only moderate discrimination and its linear additive structure cannot capture complex nonlinear interactions among renal function, contrast volume, and hemodynamics. Moreover, as a static rule-based model, the Mehran score cannot adapt to the noise, missing data, or high dimensionality inherent in electronic health records, nor does it address the extreme class imbalance of CIN, which often results in poor sensitivity in low-prevalence settings (30, 31). Recent machine learning and deep learning approaches have shown improved predictive accuracy, however, many still depend on manual feature engineering, remain vulnerable to class imbalance, and lack robustness to heterogeneous real-world data. Crucially, modern deep learning architectures designed for tabular data, particularly those leveraging self-attention to model inter-feature dependencies without handcrafted interactions, have rarely been applied to CIN prediction. Motivated by these limitations, CIN-RiskNet is designed to learn discriminative feature representations directly from structured clinical data, with SMOTE oversampling and Gaussian noise injection employed to mitigate class imbalance and simulate clinical measurement variability, respectively, while the dynamic gating mechanism suppresses noise at the feature level.

The CIN-RiskNet proposed in this study provides an important foundation for personalized treatment of PCI patients. By adopting the end-to-end TabTransformer framework, the model is allowed to learn directly from raw clinical inputs, reducing subjective factors. Our research results indicate that, compared to traditional machine learning methods, combining dynamic feature-enhanced TabTransformer with hybrid data augmentation can significantly improve the accuracy of CIN risk stratification. The core innovations and performance gains of our approach stem from the following design elements:

1. Dynamic Feature Gating Mechanism. Clinical datasets often contain many low-informative features that can mislead conventional models. Our trainable gating unit imposes feature-level sparsity constraints, adaptively amplifying informative variables and suppressing noise or redundancy. Ablation studies confirm that this module alone boosts the F1-score by 0.43% (from 0.9056 to 0.9095) according to Table 5.
2. Synergy of SMOTE Oversampling, Gaussian Noise Injection, and Five-Fold Cross-Validation. Medical data suffer from severe class imbalance and measurement noise. Our ablation study demonstrates that SMOTE combined with Gaussian noise achieves the highest F1-score (0.9542) and recall (0.9540) among all augmentation configurations, outperforming SMOTE alone (F1 = 0.9506) and noise alone (F1 = 0.9281). The Gaussian noise component simulates real-world clinical measurement variability, enhancing model robustness while SMOTE addresses class imbalance. Five-fold cross-validation ensures robust performance estimation.
3. The excellent F1 score of CIN-RiskNet suggests its strong predictive potential. In clinical practice, this model may serve as an auxiliary decision-support tool to identify patients at elevated risk prior to percutaneous coronary intervention (PCI), thereby informing personalized preventive strategies, such as consideration of aggressive hydration, low-osmolar contrast agents, or optimized procedural timing to improve renal function. Nevertheless, the efficacy of these interventions and the clinical utility of the model itself require further validation. Cautious application under rigorous monitoring is currently recommended. Compared to passive treatment after kidney damage occurs, this proactive prevention approach is more effective. At this stage, CIN-RiskNet should be interpreted as an assistive risk-stratification tool rather than an autonomous decision system. Final preventive and therapeutic decisions should remain clinician-led and integrated with bedside judgment, guideline recommendations, and institutional workflow constraints.
4. The high predictive performance of CIN-RiskNet has direct clinical implications for proactive CIN prevention. Given the insidious onset of CIN and the lack of specific post-onset therapies, accurate pre-procedural risk stratification is essential for peri-interventional renal protection.

In practice, the model can support rapid individualized risk assessment before cardiovascular intervention and help trigger preventive strategies in high-risk patients, including adequate hydration, preference for low- or iso-osmolar contrast media, strict control of contrast dose, and temporary avoidance of nephrotoxic medications when clinically appropriate.

Compared with conventional risk scoring and standard machine-learning baselines, CIN-RiskNet provides a data-driven framework for moving CIN management from reactive treatment toward earlier prevention-oriented decision support.

Going forward, we plan to collaborate with multiple healthcare institutions to build a geographically diverse dataset, allowing us to evaluate model stability across heterogeneous populations. We also aim to integrate multimodal data, combining imaging features and laboratory results to develop a comprehensive multimodal prediction system. Through the synergistic design of gating mechanisms, data augmentation, and the TabTransformer architecture, this study presents a highly accurate and interpretable CIN prediction model. While challenges remain in terms of computational complexity and cross-center generalizability, the modular innovation of our approach offers new insights for the development of medical AI models. Future efforts will focus on clinical translation and technical refinement to support the deployment of this model in real-world healthcare settings.

Several limitations should be acknowledged. First, this was a single-center retrospective study, which limits external generalizability despite the relatively large cohort (*n* = 1,679). As Transformers are susceptible to overfitting center-specific data distributions, we employed strict leakage-free stratified cross-validation, dynamic feature gating, and SMOTE restricted to training folds to mitigate this risk. Nevertheless, these internal safeguards cannot substitute for prospective multicenter validation, which remains essential before clinical deployment. Second, although we enforced strict temporal feature governance to avoid outcome leakage, independent replication in external datasets is still necessary to confirm transportability. Third, comparator analysis used a modified Mehran framework because several variables required for the original score were unavailable in our structured dataset; therefore, this comparison should be interpreted as a pragmatic local benchmark. The near-random AUROC (0.5094) reflects data incompleteness—a common real-world limitation that underscores the advantage of data-driven models capable of leveraging all available variables without requiring complete risk score reconstruction. Fourth, this study used structured tabular variables only; future work should incorporate multimodal data and prospective multicenter cohorts. In addition, future studies should include prospective impact analyses to determine whether model-guided prevention strategies can improve patient-centered outcomes beyond retrospective discrimination metrics.

## 5 Conclusion

This study proposes CIN-RiskNet, a dynamic feature-enhanced TabTransformer model with hybrid SMOTE-Noise augmentation for predicting CIN. The model significantly improves prediction performance by integrating dynamic feature selection, noise-robust data augmentation, and multi-head attention mechanisms. By unifying these components in an end-to-end architecture, CIN-RiskNet not only streamlines the workflow but also ensures that feature interactions are captured adaptively and robustly, even in the presence of noisy or imbalanced data. Experimental results show that CIN-RiskNet achieves an accuracy of 95.40%, recall of 95.40%, and F1-score of 95.42% under five-fold cross-validation, substantially outperforming both traditional machine learning models and conventional clinical scoring systems. Importantly, ablation studies confirm that each module—dynamic gating, SMOTE-Noise augmentation, and multi-head self-attention—contributes positively to overall performance gains, with the SMOTE-Noise combination achieving the optimal precision-recall balance underscoring the necessity of their combined application. Despite these encouraging internal results, routine clinical deployment is premature; prospective external multicenter validation and calibration assessment are required before broad implementation.

## Data Availability

The raw data supporting the conclusions of this article will be made available by the authors, without undue reservation.
